# The Fabrication of a Customized Surgical Template for a Miniscrew Placement Using a Fully Digitized Process

**DOI:** 10.7759/cureus.58119

**Published:** 2024-04-12

**Authors:** Li Su, Chen Luo, Hui Song, Yan P Wang, Norma Ab Rahman

**Affiliations:** 1 Orthodontics, School of Dental Sciences, Universiti Sains Malaysia, Kelantan, MYS; 2 Stomatology, Beijing Xuanwu Traditional Chinese Medicine Hospital, Beijing, CHN; 3 Stomatology, Beijing Friendship Hospital, Capital Medical University, Beijing, CHN; 4 Orthodontics, Changzhi People's Hospital, Changzhi Medical College, Shanxi, CHN

**Keywords:** digital, cam, cad, miniscrew, template

## Abstract

This report presents a clinical case involving the application of a computer-aided design and manufacturing (CAD-CAM) guide to insert miniscrew anchorage at the zygomatic alveolar ridge. A 24-year-old male adult came in with overcrowded teeth and a protruding facial profile, particularly severe overcrowding in the upper teeth and moderate overcrowding in the lower teeth. The orthodontic treatment plan involved extracting four first premolars and adding a mini-implant in the upper jaw to enhance anchorage. A miniscrew was placed in the patient's left zygomatic alveolar ridge using a guide and in the right zygomatic alveolar ridge based on experience. The use of a mini-implant guide improves the accuracy of mini-implant positioning and angulation in the infrazygomatic crest zone, reduces the risk of tooth root damage, and enhances mini-implant stability.

## Introduction

Orthodontists commonly use mini-implants as an anchorage method, and they have been widely utilized in clinical practice [1-4]. The zygomatic alveolar ridge, characterized by its abundant bone mass and double-layer cortex, provides a suitable site for mini-implant placement. Furthermore, its high position and proximity to the maxillary center of resistance make it an ideal location for mini-implant insertion. However, careful consideration of the implantation position and angle is crucial to avoid damaging vital structures such as the maxillary sinus [5-6].

This report presents a clinical case in which a mini-implant was successfully inserted in the zygomatic alveolar ridge using a computer-aided design and manufacturing (CAD-CAM) template.

## Case presentation

Diagnosis and etiology

**Figure 1:**
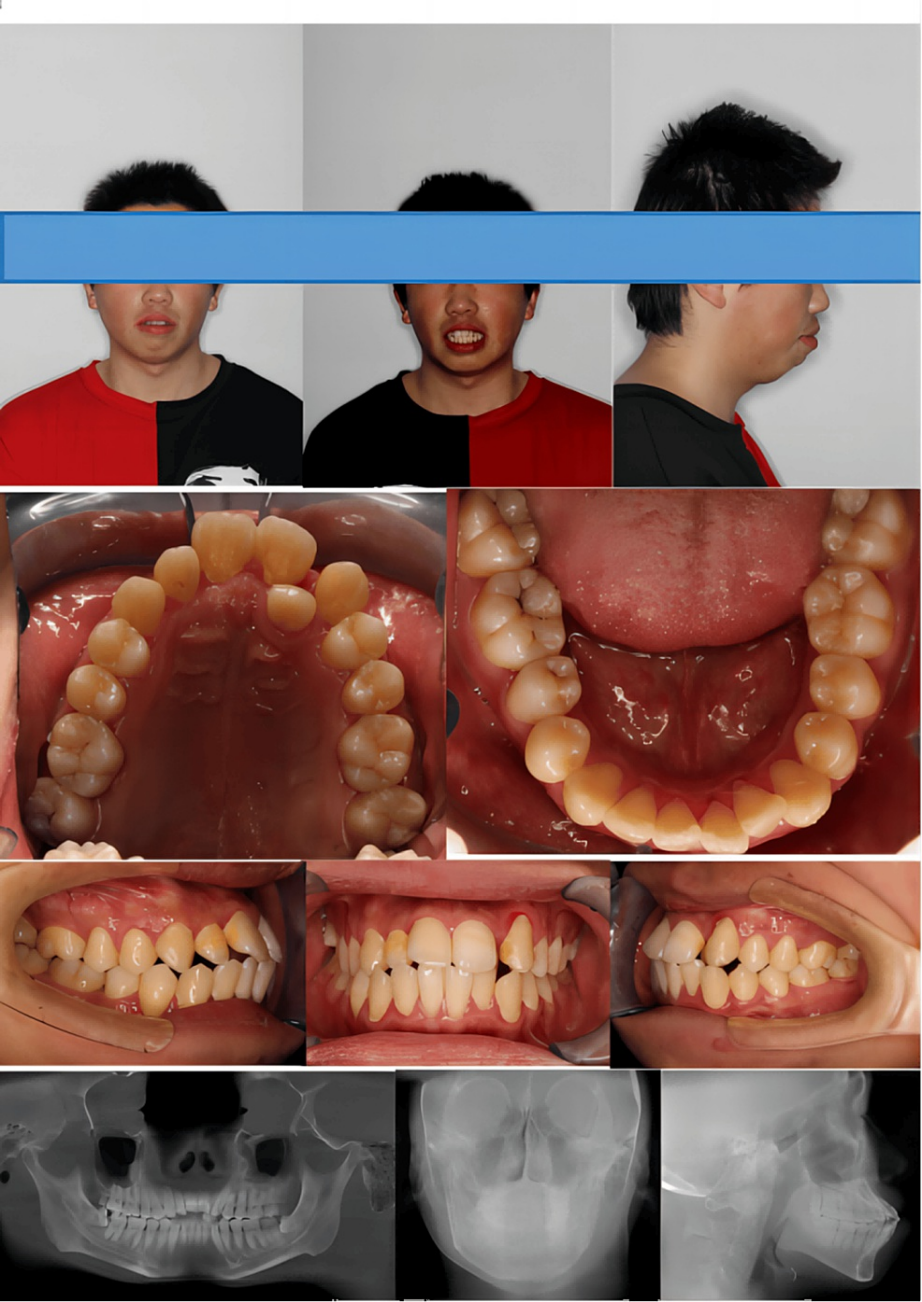
Pretreatment facial and intraoral photographs, lateral cephalogram, and panoramic radiograph

A 24-year-old adult male complained of crowded dentition and facial protrusion. The patient had no history of orthodontic treatment or trauma before this orthodontic treatment, and his periodontium was healthy. Examination revealed facial protrusion and chin retraction, with a Z-angle of around 60.75°. The patient exhibited slight facial width asymmetry, with the right side being larger than the left. There was a 5 mm deviation of the chin point to the right. The maxillary dental midline showed a 2 mm left deviation, while the mandibular dental midline exhibited a 1.5 mm deviation to the right. Severe upper arch crowding of approximately 8 mm was observed, whereas the lower arch presented a 5 mm length discrepancy. Both sides displayed class I molar and canine relationships, with 3 mm of overjet and overbite. The lateral cephalogram indicated a skeletal class II relationship (A point-nasion-B point angle (ANB), 3.13°) and a normal mandibular plane angle (sella-nasion mandibular plane (SN-MP), 36.97°). Maxillary and mandibular incisors were protruded (U1-SN: 120.25°; L1-MP, 101.94°). Additionally, the panoramic radiograph revealed two impacted mandibular third molars in elevated positions, with mild periodontal attachment loss evident. The final diagnosis included a skeletal class II relationship, dental class I malocclusion, normal mandibular plane angle, and dentoalveolar protrusion with a retruded chin (Figure 1, Table 1).

**Table 1:** Cephalometric measurements SNA - sella-nasion-A point angle; SNB - sella-nasion-B point angle; ANB - A point-nasion-B point angle; SN - sella-nasion; MP - mandibular plane; U1 - upper incisor; L1 - lower incisor

Measurement	Normal*	Pretreatment
SNA,°	81.5(1.7)	83.98
SNB,°	77.2(1.5)	80.86
ANB,°	4.1(0.9)	3.13
SN-MP,°	33.0(1.8)	36.97°
Z-angle,°	75.0(5.0)	60.75
U1-SN,°	105.7(6.3)	120.25
L1-MP,°	99.5（6.6）	101.94

Treatment objective

The treatment objective involves the safety and stability of miniscrews under the guidance of a template to prevent damage to the maxillary sinus and tooth roots.

Treatment plan

The orthodontic plan includes extracting four first premolars and adding mini-implants in the upper jaw to enhance anchorage. Mini-implants will be used to reinforce anchorage, facilitating maximum incisor retraction and profile reduction. The patient has agreed to undergo mini-implant treatment and use a CAD-CAM template.

Clinical test

**Figure 2:**
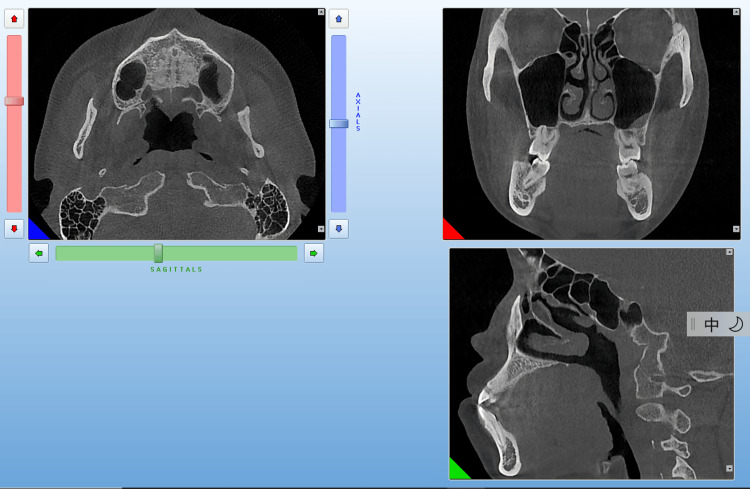
Pretreatment cone beam CT

A mini-implant was implanted in the patient's left zygomatic alveolar ridge using a guide, while another mini-implant was implanted in the right zygomatic alveolar ridge based on cone beam CT (CBCT) planning. The maxillofacial area of the patient was scanned using CBCT (Figure 2). 

**Figure 3:**
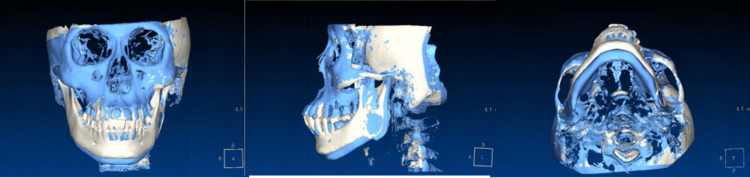
Pretreatment digital 3D model

The CBCT machine (5G, version FP; NewTom, Verona, Italy) takes CBCT films with low radiation and damage [7-8]. The CBCT's field of view is 18X16cm, and the voxel size is 0.15mm. Then the plaster model of the patient was obtained, which was necessary to ensure sufficient depth in the vestibular groove during the alginate impression. The plaster model was scanned with a D700 scanner (3shape, Copenhagen, Denmark). Both the CBCT scan data and the digital model were imported into Segma implant guide software (Beijing, China) (Figure 3).

**Figure 4:**
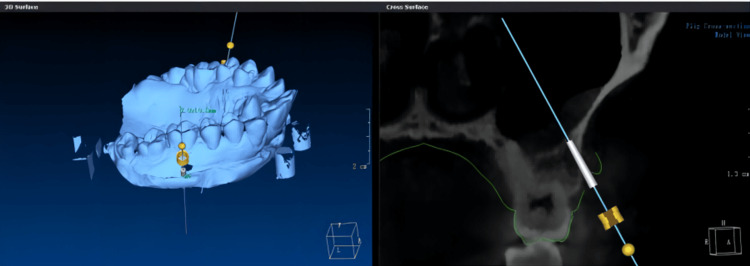
Preoperative design of the miniscrew's placement

The digital 3D model was used to design the mini-implant guide, which determined the implantation point and direction (Figure 4).

The guide was then printed using the EnvisionTEC Vida 3D printer (Gladbeck, Germany).

Surgical template design and fabrication

**Figure 5:**
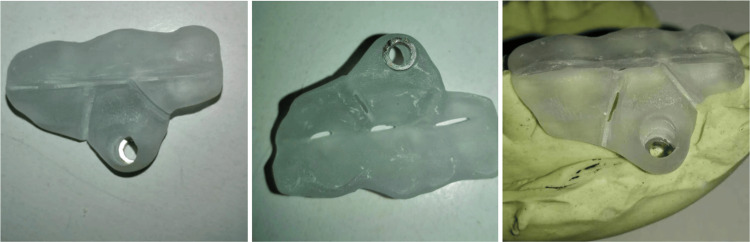
Template

As depicted in Figure 5, the proposed structure was made of three main components: a tooth guide part, a gingival guide part, and a steering part. The tooth guide and gingival guide parts are firmly connected by fracture lines. The mini-implant guide part is specifically designed to accommodate the angle and position required for miniscrew insertion.

The steering part of the miniscrew is equipped with a guide hole that accommodates a detachable limit ring. The design considerations are as follows: 1) the inner diameter of the limit ring is larger than the diameter of the miniscrew head; 2) the inner diameter of the limit ring is smaller than the diameter of the miniscrew handle head; 3) the length of the limit ring matches the length of the miniscrew minus the entry depth; 4) a marking scale is provided on the outer surface of the guide to indicate the position of the limit ring; 5) the diameter of the guide hole is larger than that of the miniscrew handle head; 6) the guide is predominantly made of resin material, whereas the limit ring is made of metal.

**Figure 6:**
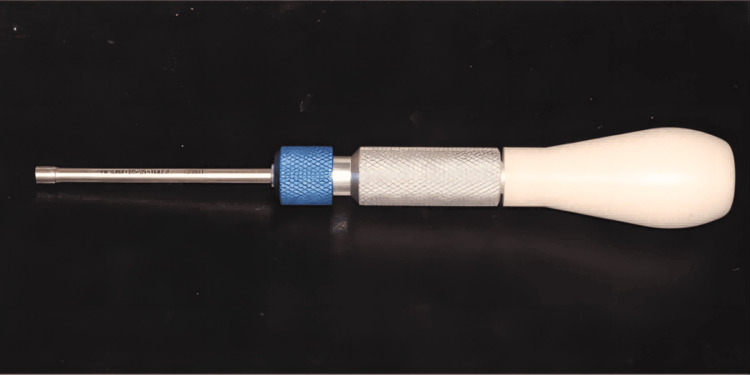
Miniscrew handle

During usage, the guide can be pre-designed based on the desired planting position of the patient's mini-implant, enabling precise guidance during the implantation process. The fracture pattern design facilitates easy fracture of the guide after the implantation of micro implant nails, simplifying the guide's detachment from the patient's mouth. A stop ring within the guide hole of the guide part effectively restricts movement and prevents damage to adjacent normal structures. The limit ring can be detached from the guide hole. Once the mini-implant is successfully implanted, the screwdriver (Figure 6) can be separated from the miniscew head by manipulating the handle.

**Figure 7:**
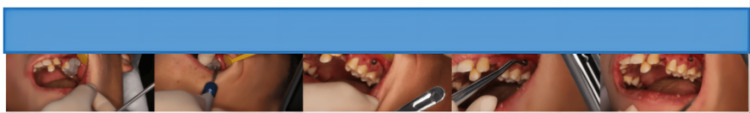
Surgical process of implanting the mini-implant with template assistance

The designed guide's data was input into the EnvisionTEC Vida 3D printer to create the mini-implant guide. Prior to the surgery, patient signs informed consent form. The implantation procedure using the guide involved the insertion of a 2 mm diameter and 13 mm length mini-implant (Cibei, Ningbo, China) into the patient's left maxillary zygomatic alveolar ridge. Upon completion of implantation, the guide and restraint ring were removed (Figure 7).

The implantation on the right maxillary zygomatic alveolar ridge was then performed by the same orthodontic professor with over 20 years of clinical experience following recommended practice. Then, the patient was bonded with Z2 brackets (Beijing, China）and coated with 0.012 nickel-titanium wire. After eight months, we applied force on the mini implant. After 18 months, we stopped applying force to the miniscrew and shot CBCT again.

Results 

**Figure 8:**
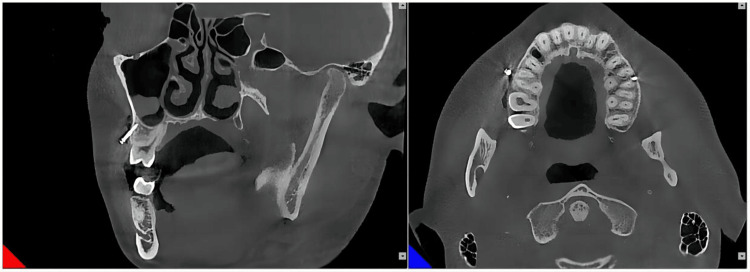
After implantation of the mini-implant in the left infrazygomatic crest

**Figure 9:**
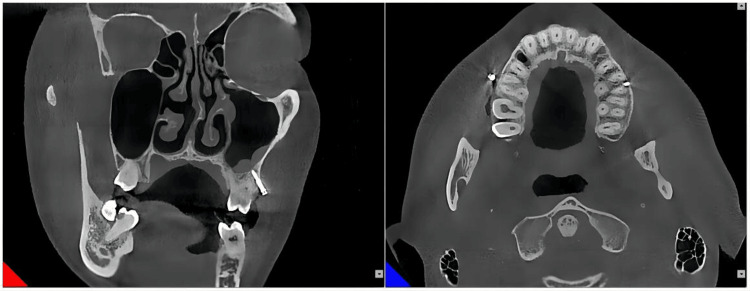
After implantation of the mini-implant in the right infrazygomatic crest

After implantation was completed, another CBCT was taken for the patient (Figures 8, 9).

The following deviations were measured: linear deviation of the mini-implant tip and cap in the bucco-palatal, mesiodistal, and vertical directions. Angular deviation of the long axis of the mini-implant in the bucco-palatal, mesiodistal, and vertical directions.

In the vertical direction, the deviations of the miniscrew tip were 0.27 mm (left side) and 1.81 mm (right side). The deviations of the miniscrew cap were 1.17 mm (left side) and 0.76 mm (right side). The angular deviations of the long axis of the mini-implant were 1.50° (left side) and 4.1° (right side).

In the mesiodistal direction, the deviations of the miniscrew tip were 0.84 mm (left side) and 1.35 mm (right side). The deviations of the miniscrew cap were 0.35 mm (left side) and 1.81 mm (right side). The angular deviations of the long axis of the miniscrew were 2.00° (left side) and 7.20° (right side).

In the bucco-palatal direction, the deviations of the miniscrew tip were 1.58 mm (left side) and 1.40 mm (right side). The deviations of the miniscrew cap were 0.44 mm (left side) and 0.24 mm (right side). The angular deviations of the long axis of the miniscrew were 5.5° (left side) and 6.2° (right side) (Table 2).

**Table 2:** Descriptive statistics of the deviations of miniscrew BP - bucco-palatal direction; MD - mesiodistal direction; VD - vertical direction

Variable	Left side (experimental group)	Right side (control group)
	Mean	Mean
			Tip at VD (mm)	0.27	1.81
Cap at VD (mm)	1.17	0.76
Angle at VD (°)	1.50	4.10
Tip at MD (mm)	0.84	1.35
Cap at MD (mm)	0.35	1.81
Angle at MD (°)	2.00	7.20
Tip at BP (mm)	1.58	1.40
Cap at BP (mm)	0.44	0.24
Angle at BP (°)	5.50	6.20

**Figure 10:**
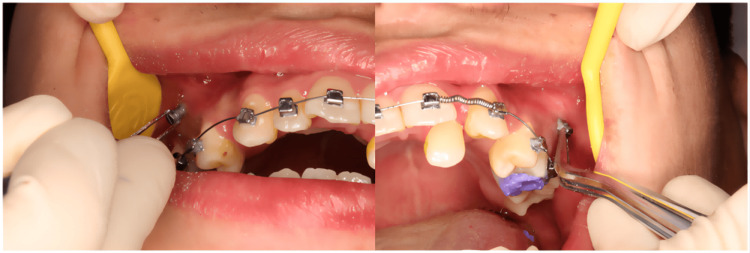
Implantation of miniscrew in the infrazygomatic crest after one month

The examination results revealed no signs of gingival redness, swelling, or loosening of the mini-implant after one month (Figure 10).

**Figure 11:**
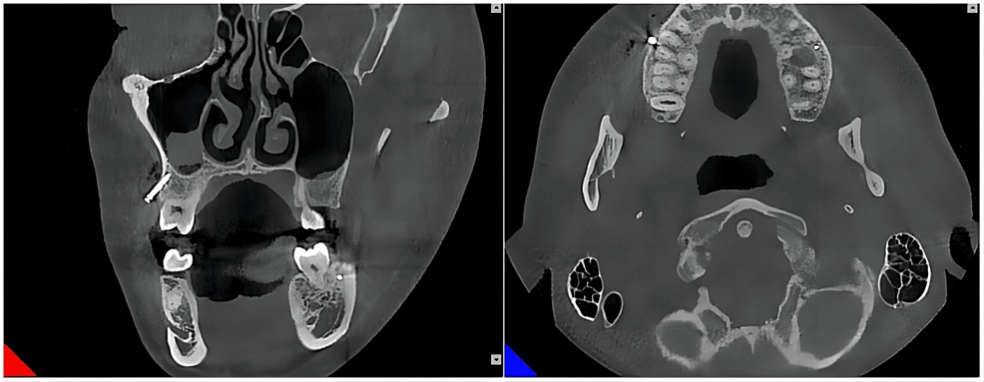
Implantation of the miniscrew in the left infrazygomatic crest after 18 months

**Figure 12:**
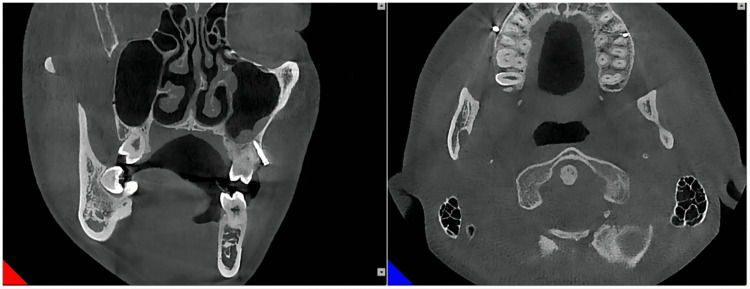
Implantation of the miniscrew in the right infrazygomatic crest after 18 months

Furthermore, after 18 months, it was observed that there was no contact between the miniscrew and the root of the tooth (Figures 11, 12).

## Discussion

Implant anchorage has become increasingly popular in orthodontics. However, a significant challenge faced in clinical applications is the risk of loosening and detachment of the implant. This issue is often attributed to limited bone mass at the implant site, which can lead to damage to adjacent tooth roots during surgery. Such damage may cause inflammation around the micro screw, compromising its stability and ultimately resulting in detachment. To enhance the safety of implantation procedures, researchers have explored various auxiliary devices aimed at improving the accuracy and safety of implantation.

In terms of the accuracy of the implant guide plate, previous studies have reported different deviation values. Bae et al. [9] found an average angle deviation of 3.14° for mini-implants implanted with the help of a guide. The average deviation of the implant cap in the mesiodistal direction was 0.29 mm, and the tip deviation was approximately 0.21 mm. Liu et al. [10], on the other hand, reported a deviation value of 0.10 ± 0.012 mm for the implantation point of mini-implants using a self-tapping guide plate. Additionally, the deviation values for the mini-implant head position, mesiodistal direction, vertical direction, and bucco-palatallingual direction were 0.42 ± 0.13 mm, 0.47 ± 0.12 mm, and 0.59 ± 0.26 mm, respectively. The deviation angles in the mesiodistal direction, vertical direction, and bucco-palatal direction were 1.2 ± 0.43°, 1.3 ± 0.41°, and 1.6 ± 0.79°, respectively.

In comparison to Liu et al.'s study, the guide demonstrated improved accuracy in the vertical direction but less accuracy in the other directions. It is important to note that our study utilized CBCT imaging and digital design throughout the entire process, specifically targeting the zygomatic alveolar ridge. On the contrary, Liu et al.'s group utilized spiral CT imaging and a guide design with lamination on the model to assist implantation between the roots of the teeth [10].

The use of a mini-implant guide can enhance the accuracy of implantation and minimize damage to adjacent normal structures. Previous research has explored the use of mini-implant guides between dental roots [11-13] and on the palate [14]. However, our study is the first to design a guide suitable for the zygomatic alveolar ridge. The zygomatic alveolar ridge provides ample bone mass and features a double bone cortex, but it is adjacent to the maxillary sinus. The research team discovered that it is safe for the implant to penetrate the maxillary sinus within a 1 mm range [6]. The objective is to ensure sufficient bone mass retention while avoiding damage to the important structures.

## Conclusions

The utilization of a miniscrew template has been shown to enhance the precision of mini-implant positioning and angulation in the infrazygomatic crest zone. The guide serves as a strategy to reduce the risks associated with potential damage to tooth roots and surrounding structures during the insertion of mini-implants. Moreover, the improved precision offered by the guide contributes to increasing the miniscrew stability. Overall, the adoption of a miniscrew guide represents a fundamental step toward improving the precision and safety of mini-implant procedures in orthodontics. In situations where conventional implantation methods pose challenges or carry a high risk of surgery-related complications, the utilization of a customized surgical template can serve as a valuable tool to aid in the placement of miniscrew.
